# Obsessive-Compulsive Training Experiences in United States (US) Medical School Education Programs

**DOI:** 10.1007/s40596-026-02360-6

**Published:** 2026-05-13

**Authors:** Carlos Valenzuela-Flores, Leanne Maduka, Dale Shepherd, Megan C. Alam, Juliana E. Avery, Hannah C. Moore, Gian DePamphilis, Megan M. Dailey, Caitlin M. Pinciotti, Andrew D. Wiese, Wayne K. Goodman, Eric A. Storch

**Affiliations:** 1 Baylor College of Medicine, Houston, TX USA; 2 COBRE Center for Neuromodulation, Butler Hospital, Providence, RI USA

**Keywords:** Obsessive-compulsive disorder, Medical education, Training, Psychiatry, Physician

## Abstract

**Objective:**

To evaluate the content and structure of OCD-specific training within United States osteopathic (D.O.) and allopathic (M.D.) medical schools. Although many physicians encounter patients with obsessive-compulsive disorder (OCD) in clinical practice, limited familiarity with the full range of OCD presentations, including typical and atypical forms, may contribute to misdiagnosis and delays in accurate diagnosis and treatment.

**Methods:**

Respondents from 35 of the 154 (22.73%) medical schools within the United States (28 (80%) allopathic, 7 (20%) osteopathic schools) completed a survey assessing demographics, school information, and OCD-focused training within medical school curriculums. Descriptives were gathered to characterize the level of training experience and type of training, and information provided throughout pre-clinical and clinical training.

**Results:**

Length of OCD-specific training varied among schools, with an average of 1.79 h total (standard deviation = 1.18 h). Few schools had faculty members or elective coursework and rotations specific to OCD training and education. Content covered was variable; for example, contamination was the most frequently taught (91.4%) while responsibility for harm was the least frequently taught (48.6%). The concept of ego-dystonia in OCD was taught in most medical schools (75.8%).

**Conclusion:**

While many medical schools contain basic education and training on OCD, there are significant gaps in training that may contribute to delays in diagnosis or even cases of misdiagnosis. Addressing these gaps in training could improve the diagnosis and treatment of OCD within clinical practice.

Obsessive-compulsive disorder (OCD) is characterized by unwanted, intrusive, and recurrent thoughts, urges, or images (obsessions) and/or the repetitive performance of behaviors or mental acts (compulsions) that are excessive or irrational and intended to curb obsessional distress [1]. Although the nature of obsessive-compulsive symptoms is heterogeneous, there are four predominant symptom dimensions including (1) contamination, (2) unacceptable/taboo thoughts, (3) responsibility for harm, and (4) symmetry [2]. While people with OCD may have varying levels of symptom insight, most experience their symptoms as ego-dystonic and/or recognize the irrationality and excessiveness [3].

OCD is relatively common with a worldwide lifetime prevalence of 1.3%, with roughly equal gender distribution of incidence [4]. Yet, it takes an average of 12.78 years from symptom onset to diagnosis which may be due in part to inaccurate diagnosis among front line clinicians [5]. In comparison, schizophrenia, another serious mental health condition with a lifetime prevalence of approximately 1%, has an average time to treatment from the prepsychotic prodromal phase of over 5 years [6, 7]. Several factors have been implicated including lack of clinician familiarity with OCD, atypical symptom presentations, other comorbid mental disorders, and limited use of validated tools [5, 8]. Among primary care physicians in the greater New York area, OCD was misdiagnosed in about half of the provided vignettes with higher rates of misidentification in taboo and harm dimensions [9]. Missed or misdiagnosis has substantial implications including delayed care, inappropriate treatment recommendations, diminished quality of life, increased disability, and financial costs for individuals and health systems [10].

Medical school curricula may provide an ideal opportunity to educate future physicians across all specialties about OCD. A study conducted among Canadian medical schools evaluated the scope of OCD-related content finding that all institutions that responded covered at least three of the symptom dimensions with a disproportionate focus on contamination and symmetry dimensions [11]. Only 25% of schools covered categories within the unacceptable/taboo dimension (i.e., aggressive, religious, and sexual), and over half of the institutions did not qualify that aggressive obsessions are ego-dystonic and do not pose risk to self or others [11]. While informative, the nature of training in United States medical schools has not been examined which was the goal of the present descriptive paper.

## Methods

All study procedures were reviewed and approved by the appropriate Institutional Review Board prior to data collection. Medical Schools with a Doctor of Allopathic Medicine (M.D.) alignment were drawn from the Liaison Committee on Medical Education (LCME), while Doctor of Osteopathic Medicine (D.O.) schools were gathered from the American Osteopathic Association [12, 13]. Inclusion criteria included accreditation from either the LCME or the Commission on Osteopathic College Accreditation (COCA). Requirements for accreditation differed between M.D. and D.O. schools and may be found on their respective websites [12, 14]. Medical schools were located within the United States of America and its territories. Those medical schools offering teaching at multiple campuses were treated as one medical school. With these criteria considered, 154 medical schools were included, comprising 126 M.D. and 28 D.O. schools.

Once a list of all eligible medical schools was sourced, representatives from each school were selected and contacted via email. Respondents were selected from pre-clinical medical education directors, psychiatry clerkship directors, and psychiatry clerkship coordinators, depending on the availability of information and access. Online surveys were sent to potential respondents at each of the 154 schools. There were no inclusion or exclusion criteria for the chosen representative besides familiarity with the respective schools’ OCD training and curriculum. Participants were informed of the purpose and type of information to be collected and were free to end participation at any time.

A 17-item survey was created by the investigative team and informed by prior research (contact authors for details on survey items) [11]. Domains assessed included school information; and OCD-specific educational and training experiences. Questions concerning school information included the name of the medical school, location, and accrediting body as well as the faculty position of the evaluator. Questions about OCD-specific training experiences included time spent on training, education on symptom dimensions (i.e., contamination, unacceptable/taboo thoughts, responsibility for harm, and symmetry/need for things to be just right), ego-dystonia, and pharmacotherapies used for the treatment of OCD. Online surveys were sent using a mail merge method to participants’ school emails. Also included in this survey was an IRB-approved cover letter introducing the study and explaining the purpose. Participants were informed that the survey was voluntary. If no response was obtained, a different representative at the medical school would be emailed. The survey was in circulation from November 2024 to February 2025.

Descriptive statistics were used to analyze responses to both demographic and interview questions. Percentages and frequencies were used to describe categorical data (i.e., type of training), while mean and standard deviation were used to describe continuous data (i.e., amount of time given to OCD training).

## Results

A total of 35 of 154 (22.73%) M.D. and D.O. training program representatives representing 19 states completed the survey, with 28 of the 35 schools (80%) accredited by the LCME and 7 of the 35 (20%) accredited by the COCA. A majority of respondents identified themselves as female (54.3%), and not of Hispanic or Latino ethnicity (85.7%). 74.3% identified as White, 11.4% as Black or African American, 8.6% as Asian, and 5.7% as other. The average age was 45.7 years. 7.9% self-identified as pre-clinical medical education directors or faculty, 65.8% as psychiatry clerkship directors, and 26.3% identified themselves as “other.” Of the 35 respondents that completed the survey, 25 (65.8%) of the interviews were completed by the psychiatric clerkship director, 3 (7.9%) by the pre-clinical medical education director, and 10 (26.3%) by another member of faculty with knowledge of the OCD training at their respective institutions.

An overview of the curriculum of each medical school revealed that formal teaching of OCD occurred only during the pre-clinical curriculum or clinical curriculum in 9 (25.71%) and 4 (11.43%) schools respectively, while 22 schools (62.86%) included formal teaching in both curriculums. Nine schools (25.71%) had a faculty member focused specifically on OCD. The amount of time devoted to the formal teaching of the clinical presentation of OCD ranged from 5 min to 5 h, with an average and standard deviation of 1.79 h and 1.18 h respectively. The most common resources used in the teaching of OCD included lectures in 29 schools (82.86%), textbooks in 23 (65.71%), and case studies in 14 (37.14%), while 3 schools (8.58%) said that students did not have access to any additional OCD-specific training resources (see Table 1).

**Table 1:** Structure of OCD curriculum at respective medical schools (*n* = 35)

	% (***n***)
OCD-focused faculty (***n*** = 35)	
Yes	25.7 (9)
No	74.3 (26)
OCD curriculum inclusion (***n*** = 35)	
Pre-clinical	25.7 (9)
Clinical	11.4 (4)
Both	62.9 (22)
OCD education tools and settings (***n*** = 35)	
Textbooks	65.7 (23)
Lectures	82.9 (29)
Simulations	11.4 (4)
Case studies	37.1 (14)
OCD-specific clinic	2.9 (1)
OCD training not accessible	8.6 (3)
Other	14.3 (5)
Time devoted to formal OCD teaching (hours), mean (***SD***)	1.79 (1.18)

Other includes workshops, online modules, and shadowing

When discussing specific training of the four primary OCD symptom dimensions (contamination, unacceptable/taboo thoughts, responsibility for harm, and symmetry/need for things to be just right) 18 schools (51.4%) reported teaching at least three of the dimensions, with contamination being the most commonly taught dimension in 32 schools (91.4%) and responsibility for harm being the least taught dimension in 17 (48.6%). When considering the types of unacceptable thoughts taught (aggressive, sexual, and religious), aggressive obsessions were most frequently taught in 23 schools (65.7%) while sexual and religious obsessions were taught similarly as often in 21 (60%). Most schools (*n* = 25; 75.8%) taught about symptoms of OCD as being ego-dystonic. All schools discussed the use of selective serotonin reuptake inhibitors (SSRIs), 30 (85.7%) of the use of tricyclic antidepressants (TCAs), and 6 (17.1%) of other treatments such as antipsychotic augmentation. Only one school (2.9%) responded that they offer OCD-related electives and coursework outside of the formal curriculum. Additional information is present in Table 2.

**Table 2:** Total percent of students taught content on obsessive-compulsive disorder (OCD) symptom dimensions, clinical presentations, ego-dystonia, and pharmacotherapies (*n* = 35)

	**% (*n*)**
Symptom dimensions (***n*** = 35)	
Contamination	91.4 (32)
Unacceptable/taboo thoughts	85.7 (30)
Responsibility for harm	48.6 (17)
Symmetry/need for things to be “just right”	71.4 (25)
Unacceptable/taboo thought presentations (***n*** = 35)	
Aggressive obsessions	65.7 (23)
Sexual obsessions	60.0 (21)
Religious obsessions	60.0 (21)
Other	8.6 (3)
None of the above	8.6 (3)
OCD pharmacotherapies (***n*** = 35)	
Selective serotonin reuptake inhibitors (SSRIs)	100 (35)
Tricyclic antidepressants (TCAs)	85.7 (30)
None	0.0 (0)
Other	17.1 (6)
Educated on ego-dystonia in OCD (***n*** = 33)	
Yes	75.8 (25)
No	24.2 (8)

Others for treatment type and Unacceptable/taboo thoughts include SNRIs and antipsychotics and post-partum OCD and somatic thoughts, respectively

## Discussion

Extended duration of illness and frequent misdiagnoses highlight a need for increased training in OCD symptom identification and diagnosis among frontline providers [5, 9]. While this issue was explored by Lahey et al. (2024) in Canadian medical schools, this is the first study to include OCD training experiences at accredited medical schools across the United States [8]. The present study assesses the current state of OCD-specific training within M.D. and D.O. schools using survey data from educational representatives from 35 medical schools across the United States. Consistent with hypotheses, data suggests that OCD curricula vary considerably across medical schools in the United States. Our findings reveal notable differences between schools in time dedicated to OCD clinical presentation, coverage of OCD domains, education on psychotherapeutic approaches, and opportunities for elective learning.

As shown in the results, the average dedicated time for teaching OCD clinical presentation was 1.79 h (SD = 1.18), with some schools allocating as little as 5 min. Eight of 35 schools provided less than 1 h of training with wide variability in the amount of instructional time. This limited exposure is likely insufficient given the complexity of OCD presentations. While contamination-related OCD was regularly taught, other common symptoms (e.g., harm or sexual obsessions) were covered in only about half of the schools. Unfortunately, these symptom presentations are common, and this training gap may directly contribute to misdiagnosis [9]. Most schools taught that symptoms are ego-dystonic, although that a quarter did not represents a significant gap in training [15]. Pharmacological interventions like SSRIs were universally covered.

The teaching methods employed may suggest concerns about depth of coverage. While lectures and textbooks were commonly used, case studies were utilized in only a minority of schools. This potentially excludes valuable context for recognizing varied OCD presentations. The relative absence of faculty specifically focused on OCD further suggests that nuanced clinical expertise may be lacking in many training environments. Few elective opportunities were reported, further limiting opportunities to develop deeper OCD expertise.

Overall, our findings parallel Lahey et al. (2024) in Canadian medical schools, suggesting that insufficient OCD training may be a widespread issue across North American medical education [11]. With high prevalence of OCD and the likelihood of encountering cases across multiple disciplines of medicine, it seems unlikely that future physicians would develop a sufficiently nuanced understanding necessary to recognize less typical OCD presentations [4]. These deficits have the potential to delay crucial diagnosis and treatment, leading to adverse outcomes in a vulnerable population. Medical educators should consider expanding curriculum time devoted to OCD in light of the incidence of the disorder, particularly focusing on varied symptom presentations beyond contamination fears, and explicitly addressing ego-dystonia as a key diagnostic feature.

Findings should be interpreted with the following limitations. First, we had a modest response rate among medical schools. It is possible that those that did not respond were less likely to have sufficient OCD coverage, and vice versa. Second, responses reflect the awareness and perspectives of individual representatives rather than objective measures of curriculum content. Future research could benefit from curriculum audits, examination of student knowledge outcomes, or assessment of diagnostic accuracy among recent graduates when presented with varied OCD presentations. Third, our survey did not specifically assess coverage of evidence-based psychotherapies like exposure and response prevention (ERP), which is a first-line treatment for OCD [16]. Despite these limitations, our findings suggest significant gaps in OCD education that may contribute, in part, to the well-documented delays in diagnosis and treatment.

This first study examining OCD education across United States medical schools reveals significant gaps in training that may contribute to delays in diagnosis and treatment. While most schools cover the basics of OCD, our findings highlight limited curriculum time (average 1.79 h, 22.9% under 1 h), uneven coverage of symptom dimensions, limited case-based learning, and few OCD-focused faculty members. Medical schools should consider expanding and diversifying their OCD curriculum to ensure graduates can recognize varied presentations of this common but frequently misdiagnosed disorder. Addressing these educational gaps could significantly improve timely diagnosis and appropriate treatment for patients with OCD.

## Data Availability

Data that support the findings of this study are available from the corresponding author upon reasonable request.
